# Machine learning for land use change analysis in environmental protection areas

**DOI:** 10.1007/s10661-026-15280-7

**Published:** 2026-04-20

**Authors:** Mayra Vannessa Lizcano Toledo, Johnnatan Rodrigues de Oliveira, Luis Armando De Oro Arenas, Leopoldo André Dutra Lusquinho Filho, Arthur Pereira dos Santos, Raphael de Vicq Ferreira da Costa, Roberto Wagner Lourenço, Darllan Collins da Cunha e Silva

**Affiliations:** 1 São Paulo State University (UNESP), Institute of Science and Technology, Av. Três de Março, Sorocaba, São Paulo 18087-180 Brazil; 2 Earth Science Institute, Pole of the University of Minho, Campus Gualtar, Braga, 4710-057 Portugal

**Keywords:** Climate, Land use change, Machine learning, Model evaluation, Vegetation

## Abstract

Anthropogenic land use and land cover change (LULCC), combined with ongoing climate variability, poses significant challenges to environmental protection areas (EPAs) by altering ecosystem structure, degrading vegetation integrity, and disrupting local climate regulation. Despite their importance, traditional LULCC approaches often fail to incorporate dynamic environmental drivers, limiting their capacity to represent complex landscape-climate interactions. This study investigates the environmental dynamics of the Guaraqueçaba Environmental Protection Area and evaluates the landscape’s potential for automated classification and prediction of impacts associated with land use change. A multitemporal dataset spanning 15 years (2009–2023) was analyzed, comprising an original set of approximately 30.2 million records of annual time series of precipitation, maximum and minimum temperatures, evapotranspiration, global solar radiation, relative humidity, wind speed, land use and land cover information, and the Normalized Difference Vegetation Index (NDVI). To address class imbalance, a balanced subset of 3.6 million records was used for modeling. Predictive models were developed using multiple linear regression (MLR), *k*-nearest neighbors (KNN), and random forest (RF), with performance assessed under both imbalanced and balanced data conditions using accuracy, $R^{2}$, precision, recall, and F1-score metrics. The results indicate pronounced local climate changes, including increasing temperatures in anthropogenically modified areas and altered humidity patterns associated with vegetation loss. Among the evaluated models, RF exhibited the highest predictive performance, achieving accuracies of up to 96% and an R$^{2}$ of 88.6%, effectively capturing the nonlinear interactions between LULCC, climate variables, and vegetation dynamics. Precipitation and NDVI emerged as the most influential drivers of LULCC processes. These findings demonstrate the effectiveness of machine learning approaches for identifying environmental degradation trajectories in protected areas and provide a robust framework to support targeted mitigation strategies and policy development applicable to other EPAs facing increasing anthropogenic pressure and climate variability.

**Supplementary Information:**

The online version contains supplementary material available at 10.1007/s10661-026-15280-7.

## Introduction

Environmental protection areas are zones categorized under Law No. 9,985/2000 as sustainable use conservation units in Brazil. This legal designation aims to recognize and safeguard these areas because of their importance for biodiversity conservation and the protection of natural resources, particularly water bodies and forests, which provide a range of benefits known as ecosystem services that ensure the environmental quality necessary for life (Maretti et al., 2023; Souza et al., 2025).

Despite this legal protection, these areas are not immune to anthropogenic pressures arising from population growth, increasing demand for natural resources, and conflicts between economic and environmental interests. When combined with inadequate enforcement, insufficient monitoring, and ineffective protective measures, such pressures cause degradation and disrupt the balance among economic, social, and environmental factors, thereby undermining sustainability (Li et al., 2024; Zhao et al., 2024).

**Fig. 1:**
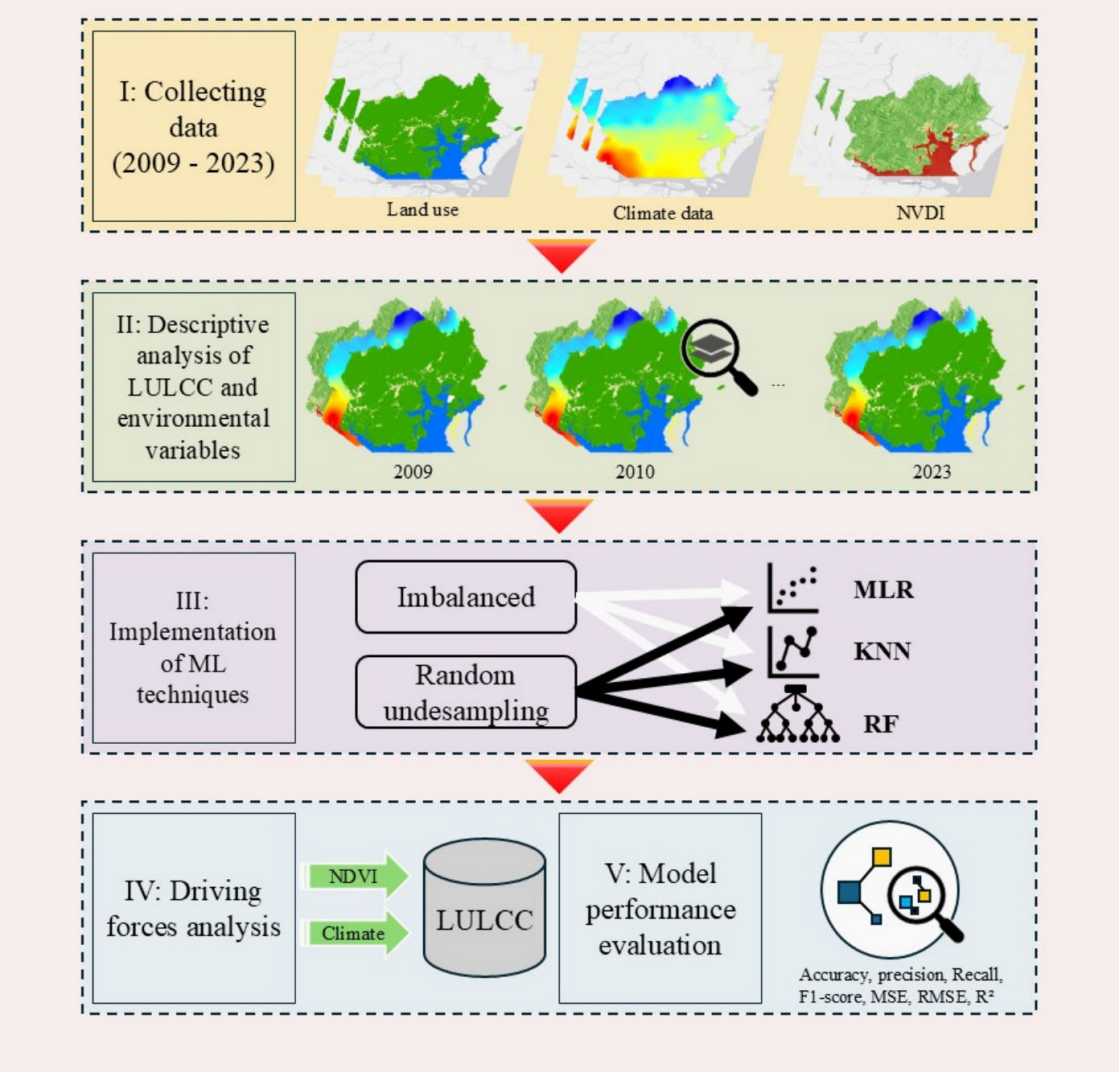
Research framework

In this context, remote sensing emerges as a tool for information analysis that supports decision-making. It is widely used to detect patterns in LULCC, agricultural expansion, the photosynthetic condition of vegetation, monitoring of fires and wildfires, and for time series analyses that contribute to studies on climate change (Ali & Jasim, 2025; Balbinot et al., 2025; Chen et al., 2024).

The combination of remote sensing with machine learning techniques represents one of the most significant advances in contemporary environmental analysis. Algorithms such as multiple linear regression (MLR), *k*-nearest neighbors (KNN), and random forest (RF) enable modeling of nonlinear relationships and identification of complex patterns in large datasets (Grinand et al., 2020; Han et al., 2023). Integrating vegetation metrics with climatic variables allows the detection of differentiated vegetation responses to environmental variability, improving understanding of the mechanisms that govern land use dynamics and their responses to climate change (Borges et al., 2025; Zarei et al., 2024). Thus, these approaches facilitate data interpretation, enhance monitoring of dynamic environmental processes, and improve prediction of future impacts, providing a basis for more effective strategies for the conservation of natural resources (Ma et al., 2024).

**Fig. 2:**
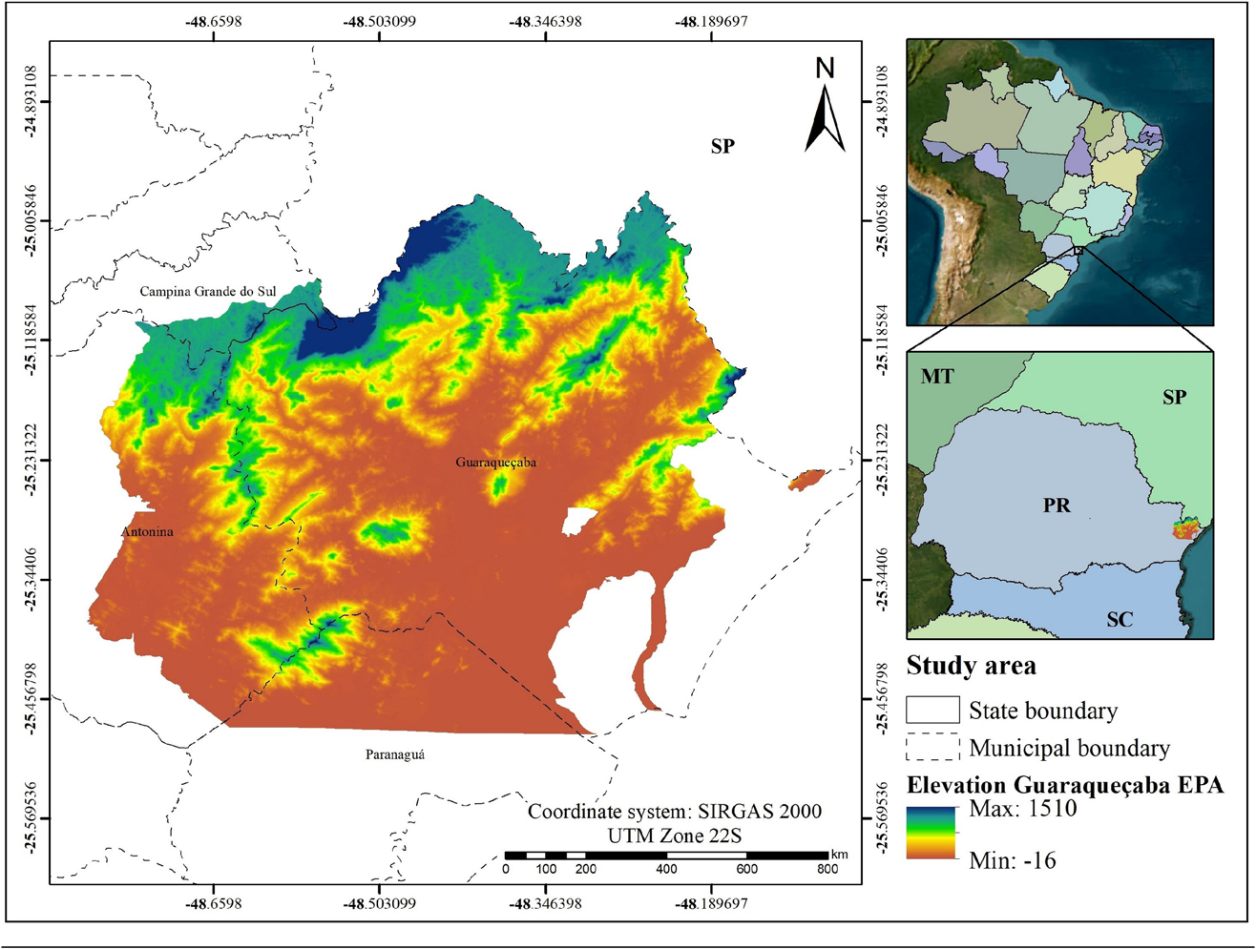
Study area

Although an increasing number of studies apply remote sensing and machine learning to analyze LULCC in Brazil, research that evaluates the relative influence of specific environmental variables over long time series and that focuses on coastal Environmental Protection Areas(EPAs), such as the Guaraqueçaba EPA, remains scarce; most work has concentrated on river basins like the Amazon (Crivelari-Costa et al., 2023), leaving a significant research gap regarding the integration of high-resolution climatic drivers with ML to model land use dynamics in Atlantic Forest EPAs.

Previous studies in the Guaraqueçaba EPA have mainly focused on point-based vegetation mapping or qualitative analyses of anthropogenic pressures, with little emphasis on quantitative modeling of land use dynamics using time series and machine learning (Bosquilia & Muller-Karger, 2021). For these reasons, the present study aimed to identify and quantify the influence of environmental variables on land use and vegetation dynamics in the Guaraqueçaba APA by analyzing time series for the period 2009–2023 using machine learning models (MLR, KNN, and RF). This study contributes to the field by explicitly addressing the role of dynamic environmental factors in a coastal conservation context. Although LULCC is widely acknowledged to be strongly influenced by socioeconomic factors, this research specifically focuses on the environmental dimension of these dynamics by examining the role of climatic variables and vegetation as factors associated with observed changes. The results are intended to provide insights that can support improved decision-making and the implementation of future strategies by public agencies, thereby increasing efficiency and contributing to the conservation of biodiversity and natural resources.

## Materials and methods

The methodological procedures followed a sequential and integrated approach (Fig. 1), beginning with data acquisition, comprising a time series from 2009 to 2023, and ensuring the spatial and temporal consistency of the information. This was followed by a descriptive analysis of LULCC and the environmental variables. Subsequently, ML techniques were implemented on both imbalanced and balanced datasets to assess the effects of class balancing on the modeling process; model performance was evaluated using various statistical metrics, and finally, an analysis of the influence of environmental variables on LULCC dynamics was conducted to identify the most relevant environmental drivers.

### Study area

The Guaraqueçaba EPA, established by Federal Decree No. 90.883/1985 (BRASIL, 1985), is located along the coast of Paraná State, encompassing the municipalities of Guaraqueçaba, Antonina, Campina Grande, and Paranaguá (Fig. 2), and covers an area of 2,826.86 km$^{2}$. The EPA is part of the Paranaguá microregion, which belongs to the Curitiba Metropolitan mesoregion, and its proximity to the Port of Paranaguá, the main port in Paraná State, should be highlighted. The microregion is characterized by a high degree of urbanization, with the exception of the municipalities of Guaraqueçaba and Morretes, where most of the population resides in rural areas (Krajevski, 2023).

The EPA is characterized by the presence of mangrove ecosystems, biodiverse estuarine systems, restinga formations, and remnants of the Atlantic Forest (Dense Ombrophilous Forest) (Pereira et al., 2025). The EPA hosts endemic species such as *Leontopithecus caissara* and *Amazona braziliensis Linnaeus*, the latter being threatened with extinction (Pereira et al., 2025). Moreover, these ecosystems play a crucial role in coastal protection, hydrological regulation, and carbon sequestration (Bosquilia & Muller-Karger, 2021; Kantek et al., 2009). The area is internationally recognized as a Ramsar site and is part of the Paraná Coast Biosphere Reserve designated by UNESCO (Mengatto & Nagai, 2022; Pigosso et al., 2018), providing habitat for numerous threatened species of flora and fauna.

From an economic perspective, the municipality of Paranaguá plays a central role, accounting for approximately 75% of the microregional GDP, whereas municipalities such as Antonina and Guaraqueçaba contribute less than 10% (Krajevski, 2023).

In recent years, the Guaraqueçaba EPA has faced increasing pressure from various sources, including agricultural expansion, predatory fishing, unregulated tourism, and illegal resource extraction, as well as the broader impacts of climate change. These anthropogenic and climatic pressures have been shown to affect vegetation cover, soil quality, and water availability, thereby undermining the composition and structural integrity of local ecosystems (Gonçalves et al., 2023).

Additionally, the lack of planning and management instruments may increase vulnerability in coastal zones, particularly in insular or isolated contexts or in areas with low socioeconomic connectivity, as observed in the municipality of Guaraqueçaba (Silva et al., 2021).

### Data acquisition and preprocessing

To assess land use dynamics and climatic variables, including maximum and minimum temperature, precipitation, relative humidity, solar radiation, wind speed, and evapotranspiration, the period from 2009 to 2023 was selected, as shown in Table 1. This time frame allows for the observation of long-term trends and interannual variability in both climatic conditions and land cover (Rendeze et al., 2018).

**Table 1:** Spatial resolution and data sources of the input variables

Variables	Resolution	Source
Precipitation		
Maximum temperature		
Minimum temperature	0.1$^\circ$ $\times$ 0.1$^\circ$	Xavier et al. (2022)
Evapotranspiration	SIRGAS 2000	
Solar radiation		
Wind speed		
NDVI		USGS
Land use classification	30 m $\times$ 30 m	MapBiomas

**Table 2:** Land use classes

COD	Classes	IBGE classes	Description
1	Water	River, lake and ocean	Includes natural or artificial
		aquaculture	bodies of water
		Forest formation	
		Mangrove	
		Wooded sandbank vegetation	They represent areas covered
2	Areas of natural	Wetland	by vegetation, including
	Vegetation	Other non forest formations	forests and other plant
		Rocky outcrop	formations
		Hypersaline tidal flat	
		Herbaceous sandbank vegetation	
		Forest plantation	
		Pasture	
		Mosaic of uses	Areas modified by human
		Soybean	activity, including urbanized
3	Anthropized zones	Rice	areas, agricultural lands and
		Other temporary crops	mining zones
		Beach, dune and sand spot	
		Urban area	
		Other non-vegetated areas	

Source: modified from IBGE (2013)

Climatic data were obtained from the dataset compiled by Xavier et al. (2022), which aggregates data from the Instituto Nacional de Meteorologia (INMET) and converts it into GeoTIFF format. These data follow international standards, such as measuring wind speed at 2 m height and air temperature at 1.2 m from the ground. Accuracy was validated using the metrics R, RMSE, MAE, Bias, CRE, CSI, and PC; results indicated moderate performance in regions with low meteorological station density, particularly the Northeast, and high performance in the central and southern Atlantic regions.

These files were reprojected to the official Brazilian coordinate system (SIRGAS2000, UTM Zone 22S). Given the original spatial resolution of $0.1^{\circ } \times 0.1^{\circ }$, inverse distance weighted (IDW) interpolation was applied to resample the data and adjust them to the same spatial resolution as the Landsat satellite imagery, ensuring spatial compatibility and accurate overlay among the heterogeneous datasets without compromising analytical consistency.

The NDVI was derived from Landsat 5, 7, and 8 level 2 imagery, accessed through the USGS EarthExplorer platform. For each year, NDVI values were calculated as the average of composites generated for the dry and wet seasons across the study area

1 $$\begin{aligned} \text {NDVI} = \frac{\text {NIR} - \text {RED}}{\text {NIR} + \text {RED}} \end{aligned}$$

where

NIR: near-infrared band reflectance

Red: red band reflectance

Land use data were obtained from the MapBiomas platform, which provides detailed land cover classifications. The original classification scheme was consolidated into broader categories (Table 2), following the Level 1 land use classification from the IBGE (2013) Land Use Manual. All classes associated with anthropogenic land use were aggregated into a single category labeled “Anthropic” for analytical purposes within the EPA context.

### Environmental variables analysis through machine learning

To explore the interdependencies between environmental variables and land use patterns, a ML-based analytical framework was implemented. This approach aimed to reveal the interactions between climatic factors and land use dynamics, as well as to identify the most influential predictors (Huang et al., 2024; Schober et al., 2018).

A suite of multivariate regression and classification algorithms was employed, namely MLR, KNN, and RF. These models were selected based on their demonstrated predictive performance and interpretability when applied to structured tabular datasets, as shown in the work of Schwartz-Ziv and Armon (2022). The modeling process began with the construction of a DataFrame in which each pixel within the study area was represented by its corresponding environmental variables and land use class, considering all years of the study period (2009–2023) jointly. In this way, each pixel-year was treated as an independent observation, allowing the capture of interannual variability in climatic conditions and land use dynamics over the 15-year period.

To incorporate categorical predictors, one-hot encoding was applied, converting categorical features into binary vectors and enabling the models to output class-probability estimates (Jagannathan & Divya, 2021). The complete multitemporal dataset, comprising approximately 30.2 million original records, was then stratified and split into training (70%) and validation (30%) sets, while a balanced subset of 3.6 million records was specifically derived for final model evaluation, ensuring robust assessment of model performance on unseen data and mitigating the dominance of common classes.

Each method was implemented twice: first using an unbalanced random sample of 5 million records and second after applying a class-balancing procedure based on random undersampling. To mitigate the dominance of the natural vegetation class, all classes were adjusted to match the size of the minority class, resulting in a balanced training dataset of approximately 2.5 million samples (representing 70% of the 3.6 million balanced subset). This strategy enhances model sensitivity to less frequent classes, particularly those associated with LULCC.

### Multiple linear regression (MLR)

MLR was employed to model the relationship between a dependent variable (land use class probability) and multiple independent environmental predictors by fitting a linear equation to the observed data Ganesh (2010). The general form of the model is as follows:

2 $$\begin{aligned} y_i=\beta _{i0} +\beta _{i1} X_1+\beta _{i2} X_2+...+\beta _{in} X_n+ \epsilon \end{aligned}$$

where

$y_i$: output corresponding to the probability of the data belonging to the land use class *i*

$$X_n$$: independent variables

$\beta _{i0}$: intercept for class *i*

$\beta _{in}$: coefficients of the independent variables

$\epsilon$: error term that captures the variability not explained by the model

The model coefficients were estimated using the ordinary least squares (OLS) method, which minimizes the sum of squared errors between observed and predicted values. To ensure that all predictors contributed proportionally to the model, data preprocessing included both scaling and balancing procedures. Scaling normalized the predictor variables to a common range, while balancing addressed potential class imbalances by ensuring that each land use class was adequately represented in the training process.

### *k*-Nearest neighbors (KNN)

The KNN algorithm was used as an instance-based method for land use analysis, benefiting significantly from the use of the FAISS (Facebook AI Similarity Search) library (Gallego et al., 2018).

The KNN model is based on the assumption that similar samples tend to be close in feature space; during the training phase, it stores all instances in the dataset and, to generate predictions, calculates the distance between a new sample and all previously observed samples, selecting the *k*-nearest neighbors (Sol et al., 2023). This approach has the advantage of being simple to understand and implement and does not require a parametric model, meaning that it does not make assumptions about the underlying data distribution.

FAISS was selected due to its high efficiency in parallel processing, particularly when deployed on GPU-supported hardware. This enabled the algorithm to manage the computational demands of identifying the five nearest neighbors within a DataFrame comprising millions of samples and seven independent variables.

The adopted distance metric was the squared Euclidean distance ($L_2^2$), which is compatible with FAISS and defined by the following formula:

3 $$\begin{aligned} d_2(x,y)= \sum _{i=1}^{n}(x_i-y_i)^2 \end{aligned}$$

The parameter *k*, representing the number of neighbors, was empirically set to 5. Various *k* values were tested to identify the configuration that achieved a satisfactory trade-off between predictive performance and computational efficiency. The feature variables were standardized to ensure equal weighting in the proximity calculations. To evaluate the relevance of the predictors in the KNN, a permutation-based perturbation method was used. For each independent variable in the test set, the values were randomly shuffled to break the correlation with the target variable while preserving the marginal distribution. The performance was then re-evaluated, and relative importance was quantified by the reduction in model accuracy. This procedure ensures that importance reflects the algorithm’s functional dependence on the attribute, avoiding common biases found in node-impurity-based methods.

This approach enabled not only a detailed evaluation of the importance of the predictor variables but also provided valuable insights into how different factors influence land use distribution, thereby optimizing resource-allocation decisions.

One of the main advantages of KNN is its ability to handle high-dimensional problems, provided that the number of neighbors *k* is appropriately tuned. However, the model also presents some limitations: as the dataset size increases, the computational cost of calculating distances between all instances grows substantially, which can lead to reduced computational efficiency. Furthermore, because KNN is sensitive to the distribution of samples in feature space, it may be influenced by noisy data and outliers, potentially reducing prediction accuracy.

Predictions were generated by averaging the values of the KNN, ensuring stable and consistent outputs, particularly in contexts characterized by complex or nonlinear data distributions.

### Random forest (RF)

Random forest (RF) is an ensemble learning method that constructs multiple decision trees to perform predictive modeling. Each tree is generated independently using a random subset of data and a random subset of explanatory variables (Salman et al., 2024). During training, trees recursively partition the dataset into increasingly homogeneous subgroups, guided by decision rules that maximize internal similarity (Sahour et al., 2021).

In this study, the target variable was defined as the land use category, and environmental variables served as predictors. The random forest model was implemented using the scikit-learn library, with explicit hyperparameter specifications to ensure robustness and reproducibility of the results. The dataset was randomly partitioned into training and testing subsets using a fixed seed (random seed = 42). The use of separate training and testing sets allows for an unbiased evaluation of model performance, ensuring that the model is not merely fitted to the training data but is also capable of generalizing to new and unseen data (Coley et al., 2023).

The model was configured to construct 100 decision trees (*n estimators* = 100). For the selection of candidate variables at each node split, the square root of the total number of explanatory variables was adopted as the criterion (*max features* = “sqrt”), following established practice in the literature. The remaining hyperparameters were kept at the library default values: maximum tree depth (*max depth* = none, no depth limit), minimum number of samples required to split an internal node (*min samples split* = 2), minimum number of samples per leaf (*min samples leaf* = 1), and the criterion used to measure the quality of a split (*criterion* = “gini”). These default settings allow trees to grow until leaves are pure or contain fewer samples than the specified minimum, thereby maximizing the model’s ability to capture complex patterns given the large volume of the dataset (30.2 million original samples, subsequently balanced to 3.6 million). As the number of trees increases, the model generally becomes more robust; however, this also entails higher computational cost and memory demand, which may represent a limitation in scenarios involving very large datasets (Lange et al., 2025).

The RF algorithm’s inherent bagging strategy contributed to reduced model variance and minimized overfitting risks (Trostianchyn et al., 2022). Variable importance was assessed using the mean decrease in impurity criterion, enabling the identification of the most influential environmental predictors. This criterion represents one of the main advantages of RF, as it provides a quantitative measure of how each variable contributes to the model’s predictive power, thereby supporting data interpretation and improving understanding of the underlying relationships.

Model training and evaluation were conducted in parallel using 20 CPU cores on a virtual machine instance (Google Cloud Computing) equipped with 128 GB of RAM and an AMD EPYC 7B12 CPU, enabling optimized processing time and efficient analysis of the large data volume.

### Metrics for model evaluation

The selection of model evaluation metrics in this study was based on the necessity to assess both the predictive accuracy and the adaptability of the models across different analytical contexts, encompassing both regression and classification frameworks. Since model outputs were interpreted as class-associated probabilities resulting from regression procedures, it was essential to employ evaluation metrics suitable for such predictive structures.

For classification tasks, accuracy was the primary metric used to quantify the proportion of correct predictions relative to the total number of predictions (Obi, 2023). To provide a more comprehensive evaluation, precision and recall metrics were also employed. Precision quantified the proportion of true positive predictions among all positive predictions, while recall measured the model’s ability to identify all relevant instances of each class (Yacouby & Axman, 2020). The F1-score, calculated as the harmonic mean of precision and recall, offered a balanced metric particularly useful in scenarios characterized by class imbalance (Takahashi et al., 2021).

**Fig. 3:**
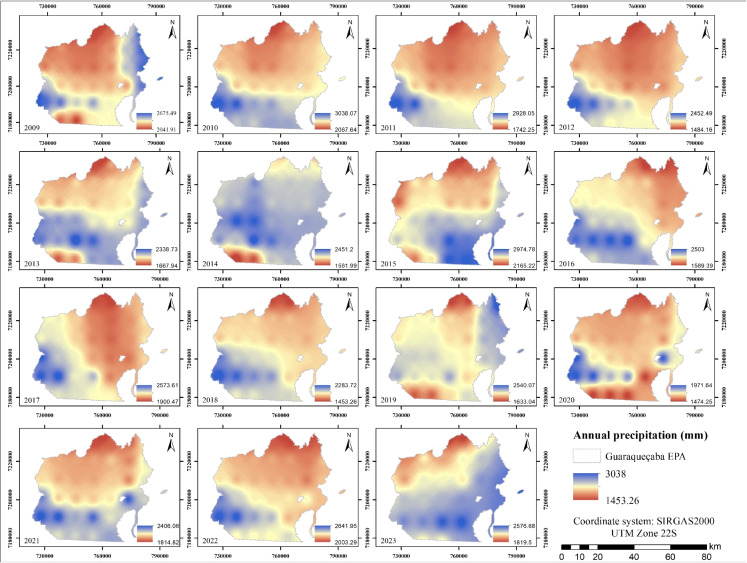
Annual precipitation maps from 2009 to 2023

Due to the pronounced class imbalance in the Guaraqueçaba EPA, where natural vegetation is the dominant class, model performance evaluation requires metrics that go beyond overall accuracy. In such contexts, class-specific metrics enable the assessment of model capability to identify less frequent yet environmentally relevant transitions. Accordingly, precision, recall, and F1-score complement global metrics by providing a more appropriate characterization of model performance in heterogeneous landscapes and protected areas.

For regression tasks, classical metrics such as mean squared error (MSE) and root mean squared error (RMSE) were utilized. MSE represents the average of squared differences between predicted and actual values, while RMSE provides a direct interpretation in the same unit as the predicted variable, facilitating intuitive understanding of error magnitude (Hodson, 2022). However, these metrics may not always fully reflect model performance. Therefore, the coefficient of determination ($R^{2}$) was also applied to measure the proportion of variance in the dependent variable explained by the model, serving as a robust indicator of its ability to capture the underlying data structure (Chicco et al., 2021).

To control the stochastic variability inherent in ML algorithms and to ensure experimental reproducibility, a fixed random seed (random seed = 42) was adopted at all stages of sampling, data partitioning, and model training. This strategy ensures that observed performance differences among the models (MLR, KNN, and RF) can be attributed to their respective architectures and data structure rather than to random fluctuations across runs. Additionally, the large volume of processed data (30.2 million original samples, with a 3.6 million balanced subset) provides high statistical stability to the performance metrics, reducing uncertainty in the estimates and rendering the application of traditional parametric significance tests unnecessary. To further enhance interpretability, Local Interpretable Model-agnostic Explanations (LIME) were utilized to provide local transparency for the instance-based KNN and linear MLR models.

## Results and discussion

### Study area environmental characterization

Figure 3 shows the annual precipitation levels in the study area between 2009 and 2023, revealing a consistent pattern with values ranging from 1453.26 to 3038 mm and an average of 2143.30 mm. Notably, the highest precipitation was recorded in 2010, whereas 2018 marked the lowest. In terms of spatial distribution, the western portion of the study area exhibits the highest rainfall, with a gradual decrease towards the east.

Additionally, Fig. 3 illustrates that areas with the highest precipitation coincide with mountainous and coastal zones. As noted by Muscarella et al. (2019), these areas are influenced by topography and the presence of moisture-laden sea winds. This robust precipitation regime is vital for maintaining the ecological integrity of mangrove and tropical forest ecosystems within the EPA (Osland et al., 2017).

**Fig. 4:**
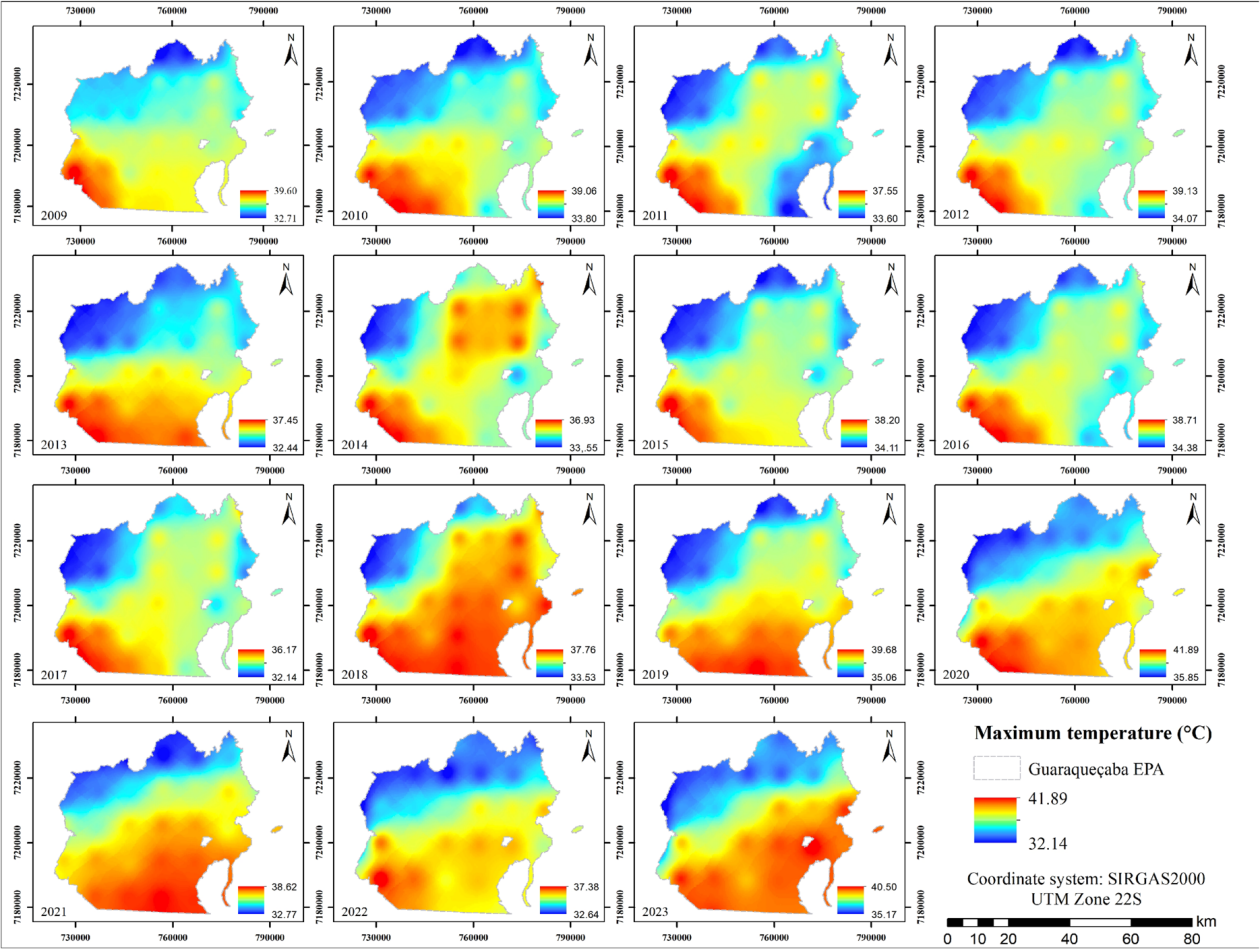
Annual maximum air temperature maps from 2009 to 2023

With regard to maximum air temperatures (Fig. 4), values across the region range from 32.14 to 41.89 $^{\circ }$C, with a mean of 36.34 $^{\circ }$C. The highest temperatures were recorded in 2020, while the lowest occurred in 2017.

Figure 4 allows the visualization of a well-defined spatial gradient of maximum air temperature, with the highest values concentrated in the southern and southeastern sectors of the Guaraqueçaba EPA, while lower temperatures predominate in the northern and northwestern portions of the region; this pattern remains relatively persistent throughout the entire time series. Some oscillations were also identified in both the intensity and the spatial extent of hotter areas, with years such as 2014, 2018, 2020, and 2023 exhibiting values close to or exceeding 40 $^{\circ }$C, whereas 2009, 2013, and 2017 presented comparatively milder conditions.

Several remote sensing-based studies have shown that areas with reduced vegetation cover or stronger anthropogenic influence tend to exhibit systematically higher maximum temperatures, whereas more conserved areas or those located at higher elevations display lower thermal values. This may be the case in the Guaraqueçaba EPA, as its northern sector corresponds to the highest elevations within the study area (Estevo et al., 2022).

**Fig. 5:**
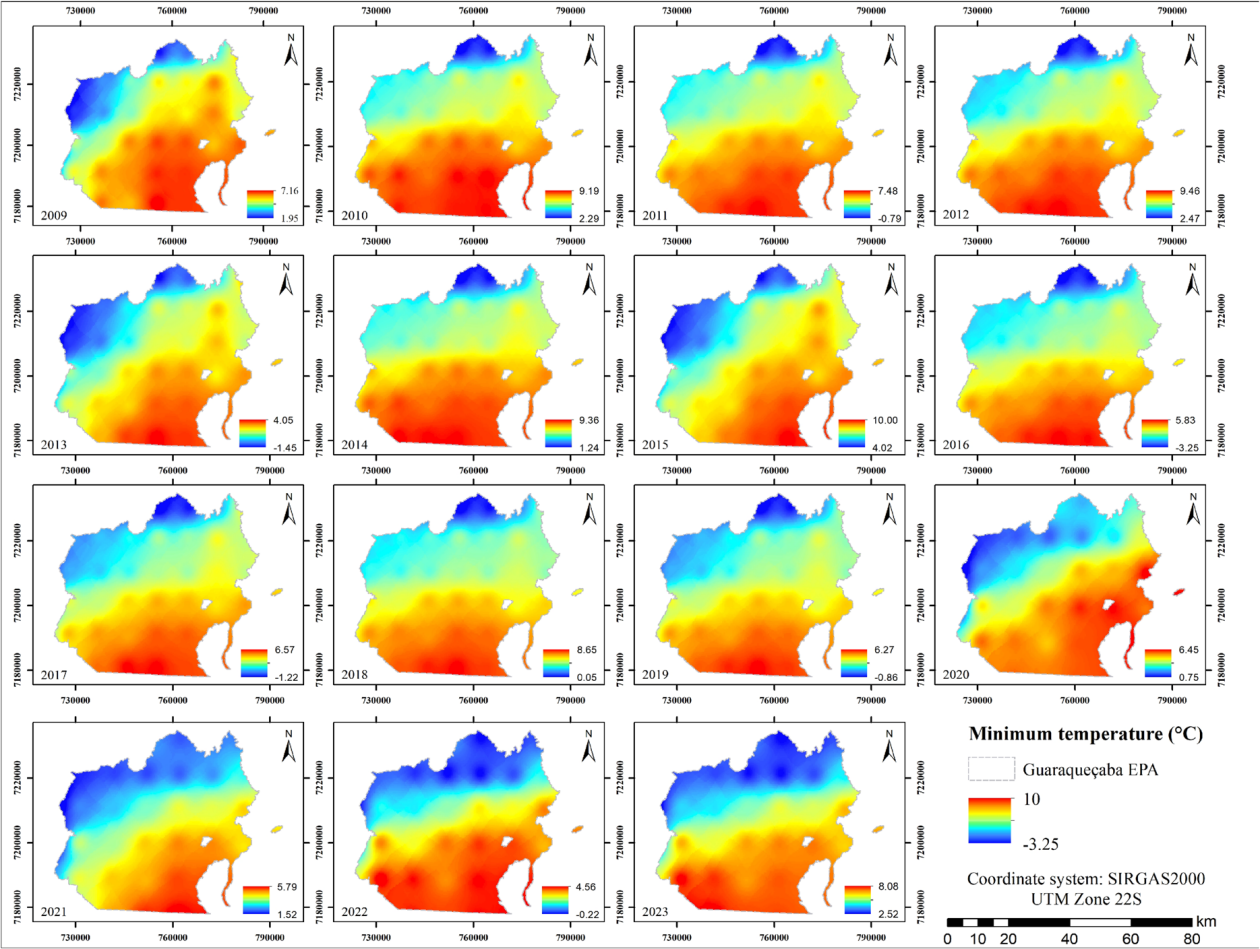
Annual minimum air temperature maps from 2009 to 2023

For minimum air temperatures between 2009 and 2023 (Fig. 5), values ranged from −3.25 to 10 $^{\circ }$C, with an average of 4.69 $^{\circ }$C. The lowest temperatures were observed in 2016, and the highest in 2015.

It was noted that vegetated areas tended to exhibit slightly higher minimum temperatures than anthropogenically altered areas. As reported by Irman et al. (2019), this phenomenon is attributed to vegetation’s capacity to retain heat during nighttime hours, thereby limiting radiative heat loss. Conversely, areas near water bodies tend to exhibit higher nighttime temperatures due to the thermal inertia of water, which releases stored heat gradually, thus mitigating nocturnal cooling in adjacent zones (Imam, 2021).

The study conducted by Gonçalves et al. (2023) identified multiple stressors and threats in the study area, including meteorological tides, storms, flooding, disruptions in biological cycles, shifts in hydrothermal and hydrological regimes, rising temperatures, land use transformation, and forest loss. The authors emphasize that climate change-related impacts may become critical if effective conservation and management measures are not implemented within the EPA.

**Fig. 6:**
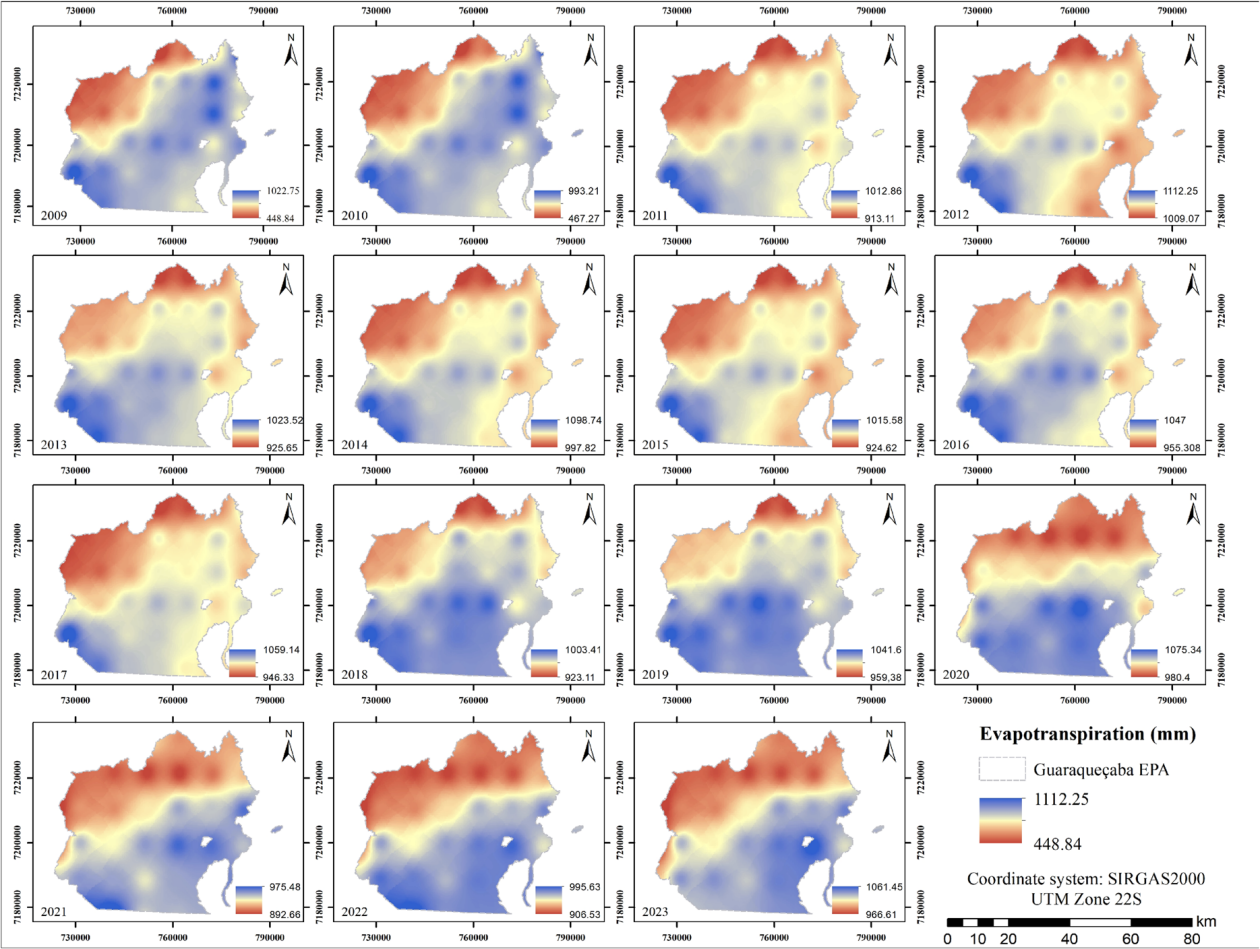
Annual evapotranspiration maps from 2009 to 2023

In terms of evapotranspiration (Fig. 6), data from 2009 to 2023 show a relatively stable spatial pattern, with values ranging from 892.65 to 1112.25 mm and an average of 990.73 mm. The highest annual values were observed in 2021, while the lowest were recorded in 2015.

The southeastern portion of the study area, which encompasses significant vegetated zones such as mangrove forests and Atlantic Forest remnants, shows the highest levels of evapotranspiration. These elevated values are associated with intensive photosynthetic activity in these ecosystems (Liang et al., 2019). In contrast, the northwestern portion exhibits the lowest evapotranspiration, corresponding to areas with greater anthropogenic disturbance and reduced vegetation cover, which limits transpiration rates and, consequently, overall evapotranspiration. These findings are essential for understanding the hydrological cycle and guiding water resource management in the region (Oliveira et al., 2021).

**Fig. 7:**
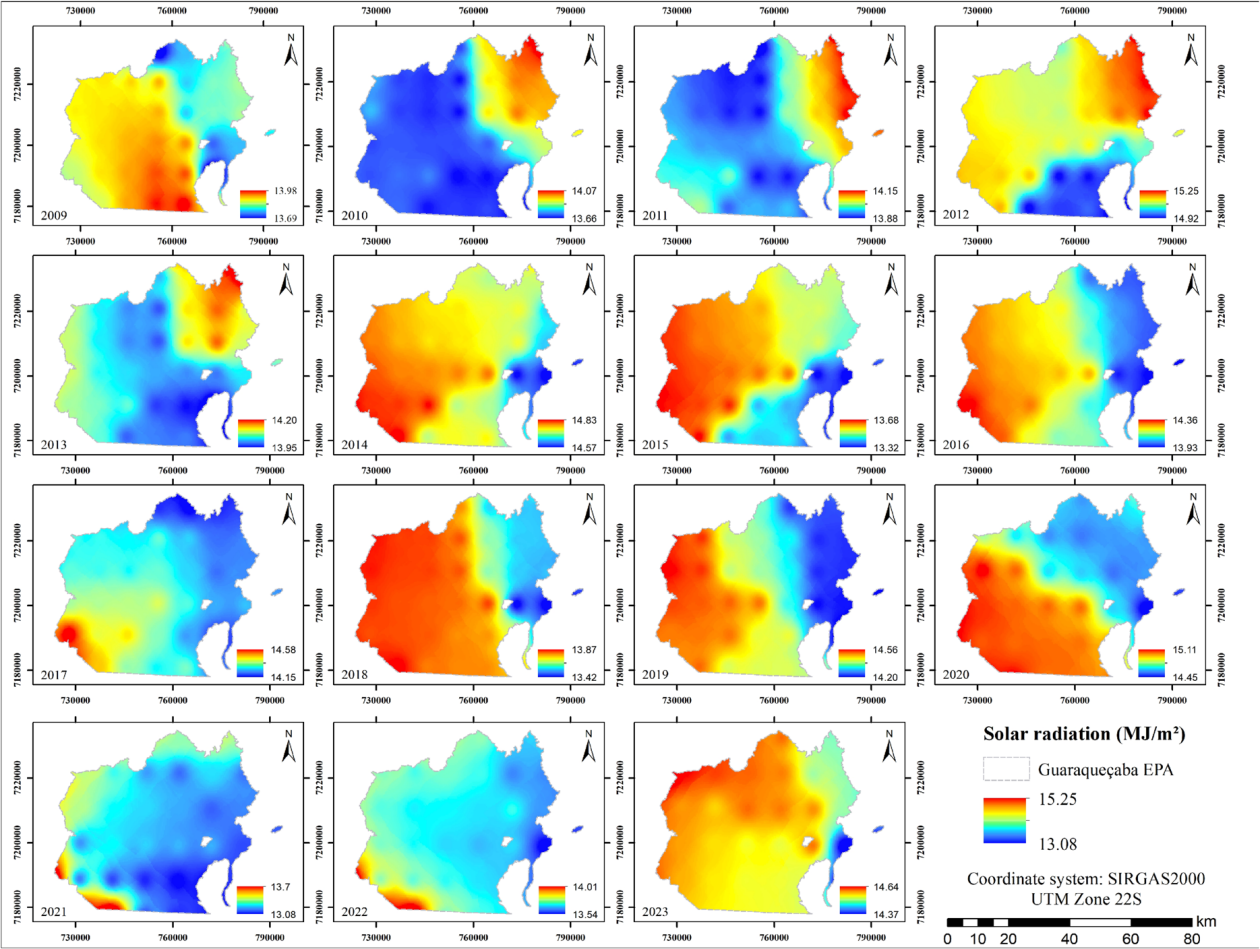
Annual solar radiation maps from 2009 to 2023

Global solar radiation received in the Guaraqueçaba EPA (Fig. 7) from 2009 to 2023 also exhibited a relatively consistent spatial pattern, with values ranging from 13.08 to 15.25 MJ/m$^{2}$ and an average of 14.13 MJ/m$^{2}$. At the beginning of the analyzed time series, the highest solar radiation values were concentrated in the northeastern sector and subsequently shifted toward the western and southern portions of the area. Considering the elevation of the EPA, topography appears to exert strong control over solar radiation, as increasing altitude may be associated with enhanced cloud formation, leading to a reduction in incoming solar radiation (Jiang & Yi, 2023).

Furthermore, areas characterized by greater anthropogenic modification, particularly in southeastern and coastal regions, tended to exhibit slightly elevated levels of solar radiation. This phenomenon is associated with the differing reflective and absorptive properties of built-up surfaces and exposed soils compared to vegetated areas, potentially resulting in localized increases in incident radiation (Nedbal & Brom, 2018).

As described by Liu et al. (2019), dense Atlantic Forest canopies intercept a significant portion of incoming solar radiation, thereby reducing the energy that reaches the surface. Moreover, vegetation albedo, which typically varies between 0.1 and 0.2, significantly influences the net radiation balance and subsequent energy partitions such as evapotranspiration. This is consistent with the known role of vegetation in absorbing solar radiation for photosynthesis and contributing to evapotranspiration. In aquatic environments, variations in turbidity and water depth can further influence the quantity of solar energy absorbed.

**Fig. 8:**
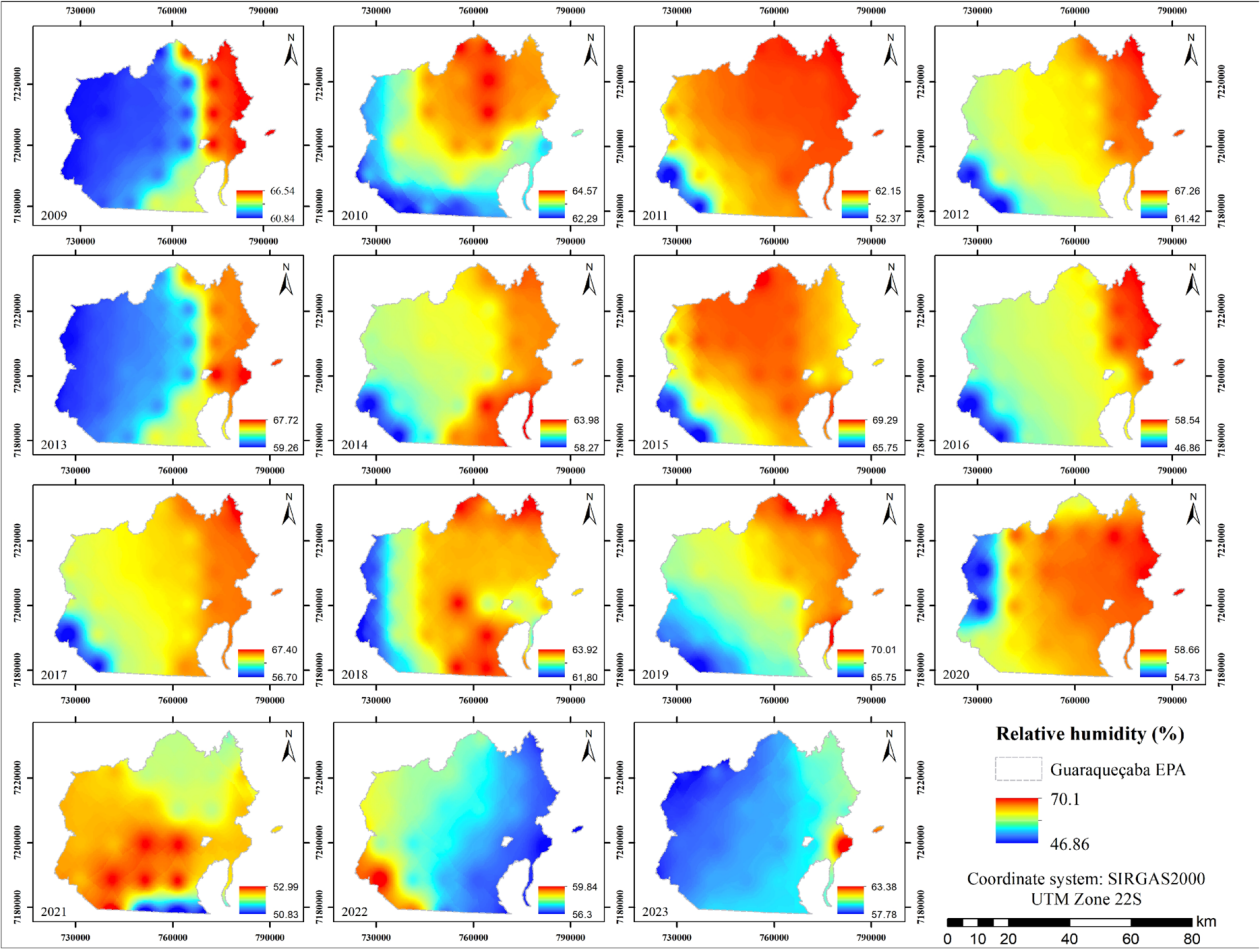
Relative humidity maps from 2009 to 2023

With respect to relative humidity (Fig. 8), values observed between 2009 and 2023 ranged from 46.86% to 70%, with an average of 61.31%. These values reflect the elevated humidity characteristic of tropical coastal regions, where the influence of the Atlantic Ocean and the presence of dense vegetation play a crucial role in sustaining high atmospheric moisture levels. Such conditions are essential for maintaining the viability of local flora and fauna.

Inland areas with a higher concentration of anthropized zones exhibited a tendency toward slightly reduced relative humidity. According to Jiang et al. (2020), this pattern can be attributed to the limited capacity of built-up and deforested surfaces to retain moisture, along with increased direct evaporation from exposed soils. These dynamics contribute to the observed reduction in local humidity (Ewer and Banks-Leite, 2013; Irman et al., 2019; Imam, 2021; Liu et al., 2023).

**Fig. 9:**
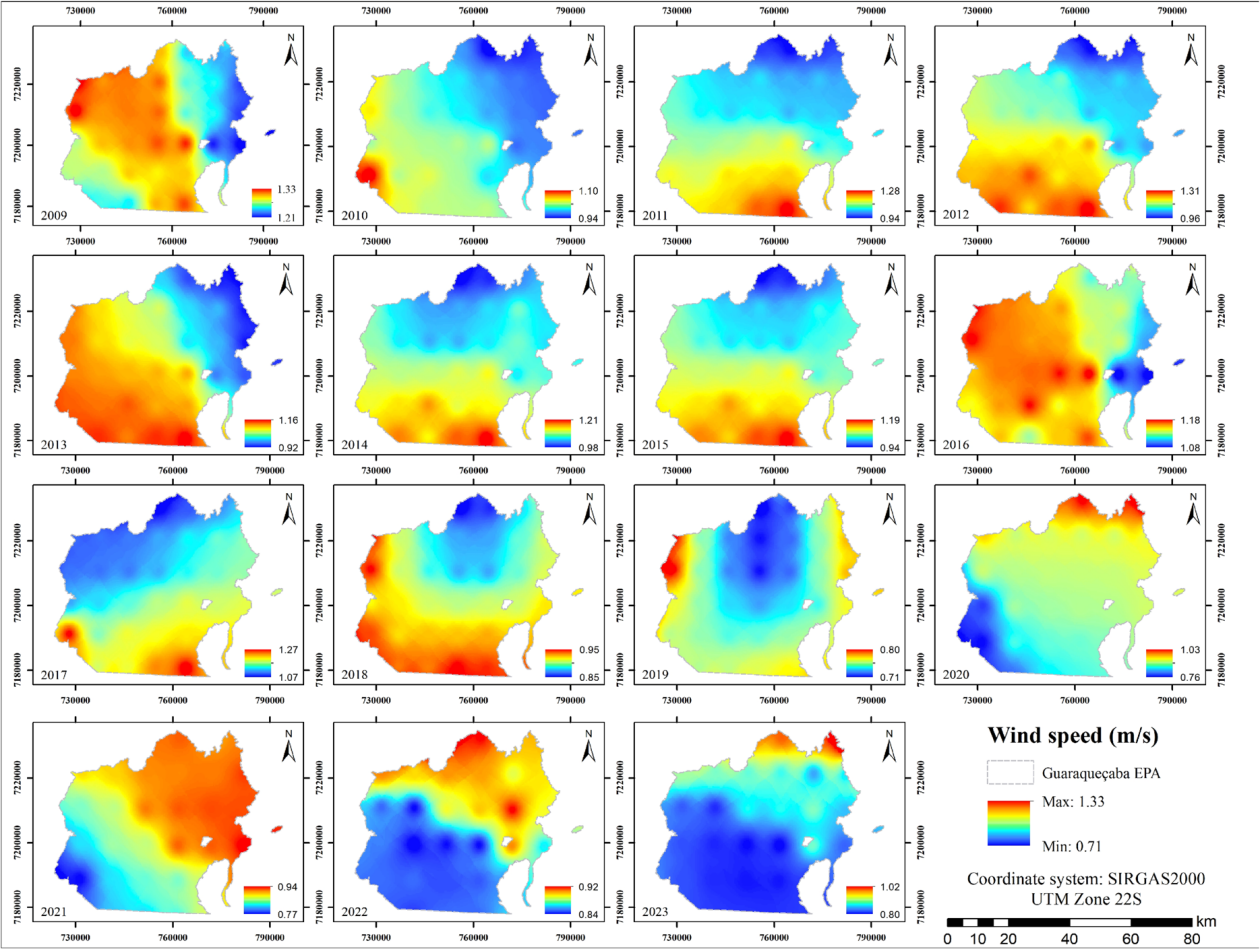
Annual wind speed maps from 2009 to 2023

Figure 9 displays the wind speed distribution over the study period, revealing values between 0.71 and 1.33 m/s, with an average of 1.01 m/s. Coastal regions experienced higher wind speeds due to the influence of trade winds and maritime air currents, while the northwestern inland sector showed comparatively lower wind speeds.

Forested areas were found to exhibit lower wind speeds, consistent with their function as natural windbreaks. The dense canopy increases surface friction and reduces wind speed near the ground. In contrast, anthropogenically modified areas, particularly those with built surfaces or sparse vegetation, tended to record slightly higher wind speeds, likely due to reduced surface roughness, which allows wind to circulate more freely (Schindler et al., 2011; Wu et al., 2018).

**Fig. 10:**
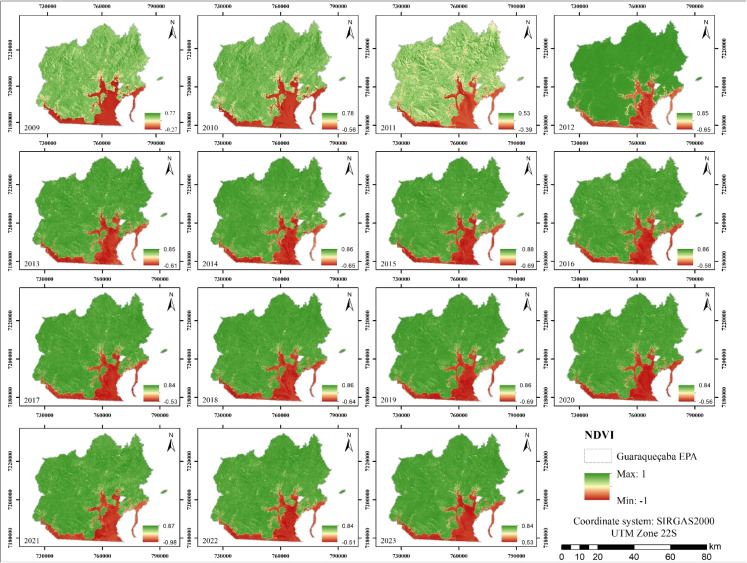
NDVI maps from 2009 to 2023

The average NDVI for the study area from 2009 to 2023 (Fig. 10) revealed elevated values in coastal zones dominated by dense Atlantic Forest and mangrove vegetation. These areas, less affected by anthropogenic pressure, reflect healthy, high-biomass vegetation. High NDVI values in such regions are widely recognized as indicators of robust ecosystem productivity and are vital for biodiversity conservation and the maintenance of ecosystem services in protected areas (Ferreira & Gurgel, 2003).

The study by Villwock et al. (2021) analyzed NDVI values from 1985 to 2009, identifying a peak in 2004 with a maximum value of 0.93, followed by a decline to 0.757 in 2009. These results are consistent with those obtained in the present study and also indicate NDVI values consistently above 0.5, which suggests the persistent presence of dense vegetation.

**Fig. 11:**
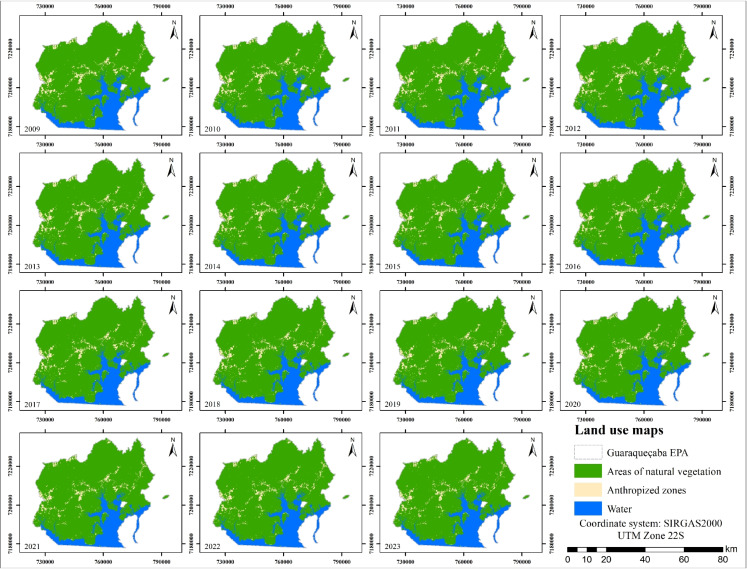
Land use maps from 2009 to 2023

Analysis of land use maps for the same period (Fig. 11) indicates progressive land cover changes, including a decline in areas of natural vegetation that were gradually replaced by anthropized zones, suggesting the expansion of human activities across the region. Nevertheless, the maps still reveal a predominance and strong persistence of natural vegetation throughout the study period, indicating that the region maintains a relatively conserved landscape structure. This pattern is consistent with the conservation status of the area, since EPAs are designed to reconcile biodiversity conservation with controlled human use.

Despite the overall stability, the maps indicate the presence of scattered anthropized zones distributed mainly in the central portion of the area and near accessible regions, associated with small settlements, agricultural activities, or other low-intensity land uses. Over time, the spatial distribution of these areas shows slight fluctuations rather than a clear expansion trend, suggesting that land use change occurs primarily through localized modifications. Such fragmentation patterns are typical of landscapes where human occupation occurs in a dispersed manner, often related to traditional or small-scale land management practices. The studies by Masolele et al. (2024); Dembélé et al. (2024) describe how such fragmentation patterns are typical of landscapes where human occupation occurs in a dispersed manner, often associated with traditional practices or small-scale land management.

**Fig. 12:**
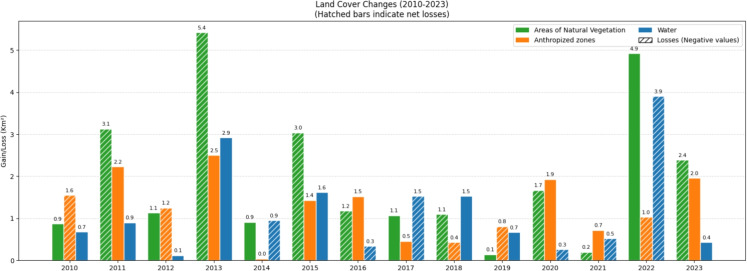
Land-use and land cover (LULC) gain-loss from 2009 to 2023

Figure 12 and Table 3 show in greater detail the dynamics of variation among land use and land cover classes in the Guaraqueçaba EPA, revealing periods marked by both losses and gains in natural vegetation. The most pronounced reductions in vegetation cover were observed in 2011, 2013, and 2015, with the first and last coinciding with an expansion of anthropogenic areas. However, despite these fluctuations and ongoing anthropogenic pressure, the Guaraqueçaba EPA still retained a predominantly conserved landscape by the end of the analyzed period.

The study conducted by Pinotti and Telles (2024) details how the municipality of Guaraqueçaba has not yet implemented the coastal management instruments established under the PNGC (National Coastal Management Plan), mainly due to the state’s low adherence to coastal management policies. The authors also highlight how peri-urban and rural communities, such as those present within the EPA, could benefit from guiding criteria for territorial planning.

According to Gonçalves et al. (2023), key drivers of land cover transformation included unregulated hunting and the selective extraction of timber and palmito (*Euterpe edulis*). Conducted without proper oversight, these practices not only induced significant economic instability but also disrupted local livelihoods. Furthermore, the introduction of invasive exotic species contributed to ecological imbalances, resource degradation, and biodiversity loss (Pigosso et al., 2018).

Additionally, artisanal fishers in the region have been promoting debates and developing proposals for the implementation of community-based natural resource management mechanisms, particularly seeking to resume traditional practices through the establishment of their own legal frameworks, emphasizing the economic, social, and cultural importance of fishing (Souza, 2023).

Conflicts have also been identified in the region involving anthropogenic practices that result in impacts such as soil compaction, wildlife displacement, the introduction of exotic species, and river siltation. Moreover, tensions exist between local populations and conservation units, leading to territorial overlap and conflicts with traditional land use and occupation regimes (Filho, 2021).

In addition, anthropogenic occupation, together with the unsustainable exploitation of crustaceans, mollusks, and fish by human populations living near mangrove areas, directly affects local biodiversity. These pressures result in the loss of feeding, breeding, resting, and life maintenance habitats, as species depend on balanced environments to sustain their populations within a given area (Pereira et al., 2025).

### Application of machine learning to different scenarios

Following the training of the MLR, KNN, and RF models, performance results were obtained as summarized in Table 4, which presents each model’s evaluation under both imbalanced and balanced data scenarios. These results provide a detailed overview of the models’ capabilities, limitations, and appropriateness for addressing the complexity of land use classification tasks.

The performance metrics presented in Table 4 reflect the predictive capability of the models over the multitemporal dataset covering the entire study period (2009–2023), incorporating interannual variability in climatic conditions and LULCC dynamics. The comparison of the results shown in Table 4 indicates that high accuracy values may coexist with substantial limitations in identifying less frequent classes. This behavior is particularly evident in the MLR model, in which global accuracy does not adequately reflect class-level performance. In contrast, the RF model shows greater consistency between global and class-specific metrics, indicating a more robust representation of land use transitions in a landscape dominated by natural vegetation. The convergence observed between regression metrics (R$^{2}$ and MSE) and classification metrics (accuracy and F1-score), together with the large volume of data analyzed, suggests that performance differences among models are stable and reflect their structural capacity to capture land use dynamics over the analyzed period, rather than random variations in the training process.

**Table 3:** Area by LULC Class from 2009 to 2023

Category	2009	2010	2011	2012	2013	2014		
Water	456.29	455.611	454.71	454.59	451.67	452.62		
Areas of natural vegetation	2256.81	2255.93	2259.06	2257.93	2263.35	2262.44		
Anthropized zones	113.77	115.32	113.09	114.34	111.84	111.81		
2015	2016	2017	2018	2019	2020	2021	2022	2023
451	451.33	452.86	451.32	450.65	450.92	451.43	455.33	454.9
2265.48	2266.66	2265.6	2266.69	2266.56	2268.23	2268.43	2263.5	2265.89
110.38	108.86	108.41	108.84	109.65	107.72	107	108.02	106.07

**Table 4:** Performance metrics in percentages (%) of regression models

	MLR		KNN		RF	
	Imbalanced	Balanced	Imbalanced	Balanced	Imbalanced	Balanced
Accuracy	81.05	48.60	89.75	86.35	96.34	95.26
Precision	72.01	45.79	89.17	86.50	96.26	95.38
Recall	81.05	48.60	89.75	86.35	96.34	95.26
F1-score	72.62	45.31	89.37	86.06	96.25	95.21
MSE	9.67	19.78	4.92	6.63	1.96	2.53
RMSE	31.09	44.48	22.17	25.76	14.00	15.92
$R^2$	7.14	10.99	48.03	70.15	77.89	88.60

In the case of the MLR model under the imbalanced scenario, a global accuracy of 81% may initially suggest satisfactory performance. Nevertheless, in imbalanced datasets, traditional metrics such as accuracy lose reliability, as correct classification of the majority class may mask systematic errors in minority classes. When complementary metrics are considered, such as precision and F1-score (both at 72%), it becomes evident that the model struggles to maintain a balance between sensitivity and specificity. The equivalence between accuracy and recall indicates a highly sensitive model but with low specificity, particularly in classifying the minority classes “Anthropic Areas” and “Water” (Ballabio et al., 2018). Additionally, the high MSE (9.67%) and RMSE (31%) values indicate substantial error margins in individual predictions, while the low $R^{2}$ value (7.14%) confirms the limited ability of the linear model to explain the total variability of the dataset.

When applied to a balanced dataset, the performance of the MLR model declines further, with accuracy dropping to 48%. This reduction highlights its limited ability to address class-equitable data distributions. Precision (45%), recall (48%), and F1-score (45%) collectively demonstrate a uniform degradation in predictive quality, which is consistent with the inherent limitations of linear models in representing complex, nonlinear relationships. The MSE (19.78%) and RMSE (44.48%) increase significantly, reinforcing the model’s instability. Although the $R^{2}$ slightly improves to 10.99%, this marginal gain is insufficient to compensate for the broader performance losses. As emphasized by Kim (2019), these results confirm that MLR is more appropriate for identifying simple linear dependencies and is ill-suited for modeling the intricacies of heterogeneous or balanced environmental datasets.

The KNN model demonstrated greater robustness and flexibility across both scenarios. In the imbalanced dataset, KNN achieved an accuracy of 0.89, with weighted precision, recall, and F1-score all at 89%, suggesting well-balanced performance. These results indicate the model’s capacity to classify both majority and minority classes effectively. The MSE of 4.92% and RMSE of 22.17% reflect relatively low error margins, and the $R^{2}$ of 48.03%, though not optimal, is markedly superior to that of MLR, revealing KNN’s effectiveness in modeling nonlinear relationships.

When evaluated using a balanced dataset, KNN exhibited a slight reduction in accuracy (86%) and recall (86%), while maintaining high precision (86%) and a consistent F1-score (86%). This consistency underscores the model’s adaptability to variations in class distribution. While MSE and RMSE increased modestly to 6.63% and 25.76%, respectively, the $R^{2}$ improved significantly to 70.15%, indicating enhanced explanatory capacity in a more challenging scenario. Variable importance analysis identified minimum temperature (Tmin) as one of the most influential predictors in the balanced scenario, suggesting that the KNN model is particularly responsive to climatic variables under these conditions.

The RF algorithm produced the most favorable results in both data scenarios. In the imbalanced dataset, RF achieved an accuracy of 96%, with precision, recall, and F1-score all consistently at 96%. This performance reflects excellent classification balance. The MSE (1.96%) and RMSE (14%) were the lowest among the models, and the $R^{2}$ of 77.89% confirmed the model’s high explanatory power, demonstrating its ability to account for the majority of data variability. However, as noted by Lei et al. (2004), the superior performance in the imbalanced scenario may be partially influenced by the dominance of the majority class, despite the uniformly strong precision and recall values.

In the balanced scenario, RF maintained a high accuracy (95%) and achieved an $R^{2}$ of 88.6%, representing a slight improvement over the imbalanced case. These results indicate that RF effectively adapted to the balanced class structure while extracting more detailed information from the predictor variables. The MSE (2.53%) and RMSE (15.92%) remained low, further reinforcing RF’s status as the most reliable and efficient model among those evaluated.

This level of performance is consistent with the advantages of ML-based approaches over traditional land use simulation methods, such as CA-Markov models, which commonly report global accuracies between 0.80 and 0.90 and rely on transition matrices that assume temporally stationary probabilities (Asif et al., 2023; Zhang et al., 2023). Unlike these stochastic frameworks, which primarily propagate historical patterns, the RF model was able to capture nonlinear feedbacks and the direct influence of climatic (e.g., precipitation) and biophysical drivers (e.g., NDVI) at the pixel level, a capability that is particularly relevant in protected areas where land use dynamics are sensitive to abrupt environmental variability (Vahid & Aly, 2025; Wang et al., 2022; Zhang et al., 2023).

Several studies, such as those by Anees et al. (2024, 2025), demonstrate the effectiveness of combining RF with remotely sensed data analysis, highlighting its high predictive accuracy. These studies report promising results when applied to time series data, and Anees et al. (2025) further observed superior performance compared to the XGBoost model.

An expanded analysis of class imbalance indicates that undersampling was an effective strategy for the random forest model, allowing improved extraction of information from predictor variables and increasing $R^{2}$ from 77.89% in the imbalanced scenario to 88.60% in the balanced scenario. However, alternative mitigation strategies warrant consideration. The use of class weights could preserve the full data volume, while synthetic oversampling techniques, such as SMOTE, could generate additional samples for the anthropic class. Nevertheless, in spatial analyses of protected areas, such approaches may preserve structural bias toward the majority class or introduce artificial patterns. Thus, the adopted undersampling strategy represents a methodological compromise among bias reduction, ecological interpretability, and preservation of the real spatial structure of land use transitions, avoiding inflated accuracy that would merely reflect the dominance of native vegetation.

**Fig. 13:**
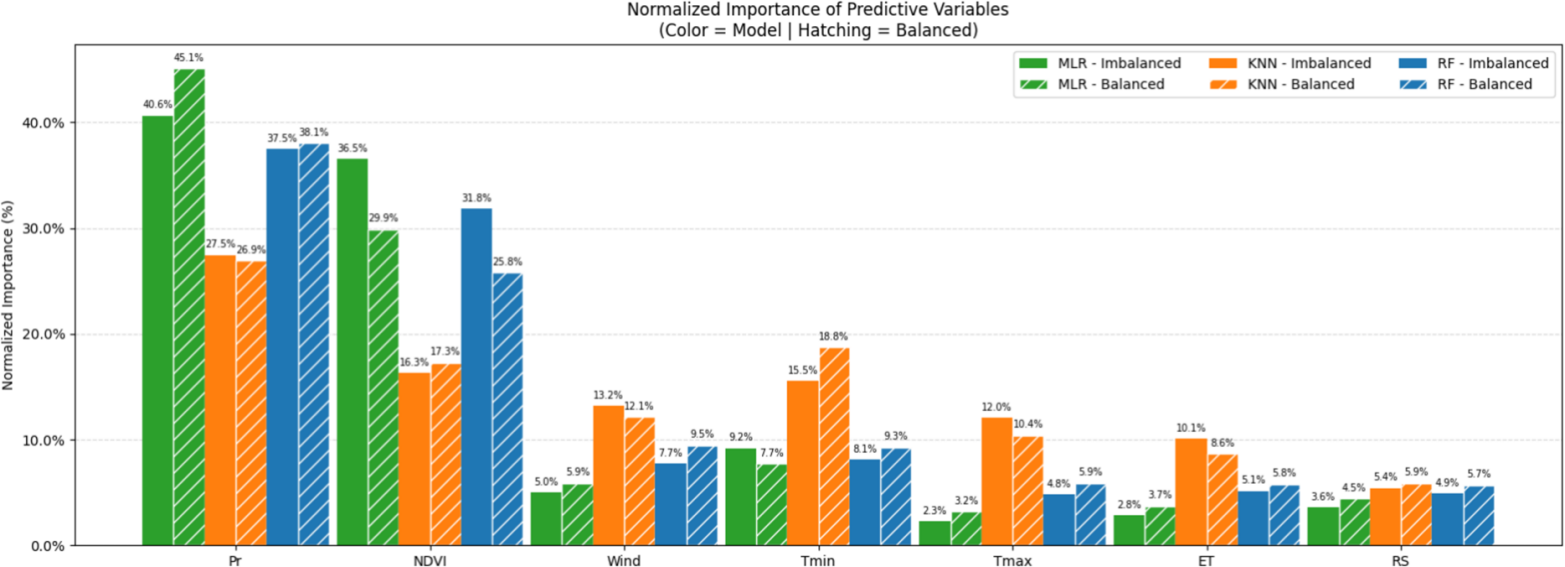
Normalized importance of predictive variables for MLR, KNN, and RF models

To complement the analysis of model robustness and enable direct visual comparison among the tested algorithms, Fig. 13 presents the normalized importance of predictor variables for each model, detailing the relative influence of each climatic and vegetation variable and contrasting model sensitivity under both the original (imbalanced) and undersampled (balanced) scenarios.

Regarding the influence of the variables, as shown in Fig. 13, it is evident that their relative importance varies depending on the model and the data scenario. In the imbalanced MLR model, the Normalized Difference Vegetation Index (NDVI) (36.55%) and accumulated precipitation (Pr, 40.61%) emerged as the most influential predictors, whereas wind speed (4.98%), minimum temperature (Tmin, 9.18%), and solar radiation (RS, 3.57%) demonstrated lower relative importance. In the balanced scenario, although Pr (45.1%) and NDVI (29.89%) remained the most relevant, the relative importance of wind speed (5.88%) and evapotranspiration (ET, 3.74%) increased, suggesting that, in this context, atmospheric and climatic variables exert a greater influence.

Across nearly all models, accumulated precipitation and NDVI were consistently identified as the most significant predictors. This finding aligns with ecological theory, as water availability and vegetation condition are key determinants of LULCC (Mo et al., 2019). The analysis in Fig. 13 also reveals that KNN attributes a more distributed importance among predictors compared to RF. This occurs because the permutation method used in KNN evaluates global neighborhood loss, highlighting that variables such as Tmin gain critical relevance (18.8% in the balanced scenario) by influencing the local proximity structure between pixels, a relationship that linear metrics (MLR) fail to capture. Variables such as wind speed, maximum and minimum temperatures, solar radiation, and evapotranspiration showed variable importance depending on the model used and whether the data were balanced or imbalanced. This variability reflects their more subtle or indirect roles in driving LULCC, suggesting that the relevance of such variables may be conditional upon the distribution of other environmental factors.

The study by Mehmood et al. (2025) highlights the strong influence of factors such as temperature and precipitation in forests, as they can induce water stress that leads to degradation; it also emphasizes that these variables should be considered when developing forest management strategies aimed at increasing resilience to extreme climatic events.

In the MLR model, NDVI was identified as the most important variable in the imbalanced scenario, followed by precipitation. This suggests that the linear relationships between water availability and vegetation greenness explain a substantial portion of the modeled response. In the balanced scenario, however, wind speed and evapotranspiration gained prominence, indicating that class redistribution exposes the influence of variables associated with less dominant land use types. The predominance of precipitation reflects its direct role in controlling soil moisture and plant productivity, while NDVI acts as an integrative indicator of vegetation condition that encompasses multiple environmental factors.

**Fig. 14:**
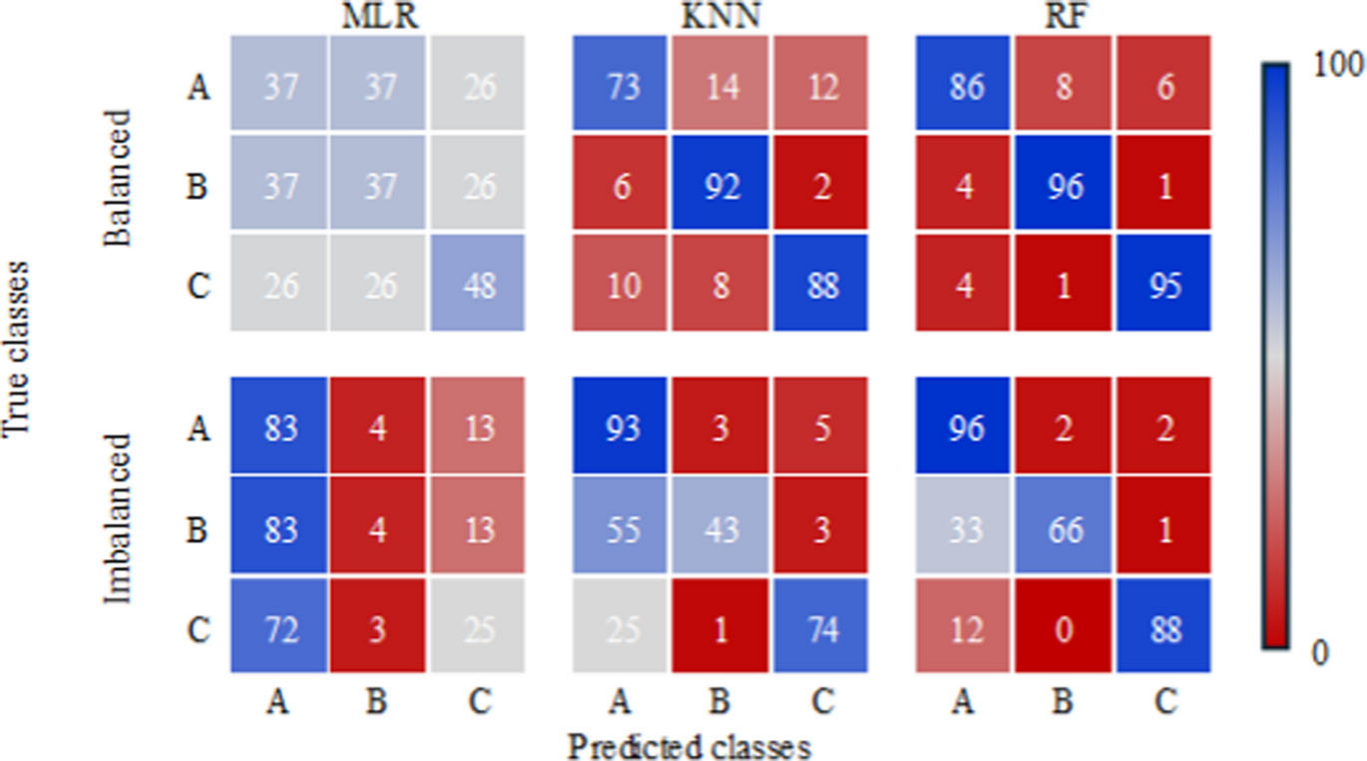
Confusion matrices for MLR, KNN, and RF models under both original (imbalanced) and undersampled (balanced) scenarios

In the case of KNN, importance was distributed more evenly among predictors, with notable contributions. Tmin played a critical role in the balanced data, while Pr and NDVI retained their importance in both scenarios. This behavior demonstrates the model’s capacity to detect nonlinear interactions and adapt effectively to varying class distributions. Furthermore, it indicates the relevance of localized, nonlinear interactions in which thermal constraints and atmospheric dynamics influence system behavior.

For RF, the central role of Pr and NDVI is reinforced, but the model also revealed secondary contributions from wind and temperature variables, highlighting the ability of tree-based models to capture complex interactions and hierarchical effects not represented in linear formulations. In addition, the model also identified Tmax, RS, and ET as key contributors to land use and land cover dynamics (Audia et al., 2020; Li et al., 2017). These results underscore RF’s robust ability to recognize complex patterns and interdependencies within the data, even under scenarios characterized by high variability. Consequently, RF not only outperformed other models in terms of predictive metrics but also proved the most comprehensive in identifying relevant predictors across both imbalanced and balanced datasets (Lei et al., 2004).

Given that approximately 81% of the EPA’s land cover is classified as natural vegetation, any model predicting this dominant class inherently has a high likelihood of achieving correct classification. This phenomenon is especially prominent under imbalanced conditions, where high accuracy may be accompanied by a low $R^{2}$ value, particularly in models such as MLR that exhibit weaker performance and limited ability to capture the variance within the dataset.

The study by Zeferino et al. (2020) used the random forest model to investigate the influence of environmental data such as NDVI, precipitation, and temperature on the accuracy of LULCC classification; the results indicated that these variables have a significant impact on LULCC dynamics, showing that their incorporation into the model substantially increases prediction accuracy.

Figure 14 illustrates the confusion matrices for each of the three models under both data scenarios, revealing how each land use class was predicted.

The MLR matrix highlights how, in the imbalanced scenario, there is a strong tendency to classify the three classes as natural vegetation areas, exhibiting high commission errors and low accuracy for anthropogenic areas and water. This may be explained by an insufficient amount of training data for the model to capture the characteristics of class B in particular. In the balanced scenario, the model shows partially improved performance in classifying class B, with better accuracy for this class as well as for class C compared to the imbalanced scenario; however, this is accompanied by a decrease in accuracy for class A.

Conversely, the imbalanced KNN scenario presents a more notable balance among all classes, with errors more distributed and less frequent; again, class B shows the largest commission errors, which may be caused by the smaller number of training samples, given its limited extent within the Guaraqueçaba EPA. On the other hand, in the balanced scenario, the model is able to adjust its classifications to better capture variability among classes, maximizing its ability to identify local patterns.

Finally, the RF matrix demonstrates the model’s superiority, with better performance in the imbalanced scenario compared to the other two models, showing improved classification capability for class B, although still with 33% of data misclassified. In the balanced scenario, RF exhibits high accuracy with low presence of commission and omission errors for each of the analysed classes, where more than 85% of the data were correctly classified for each class, reflecting the model’s strong performance due to the greater uniformity of sampling in the balanced scenario.

### Limitations and suggestions for future work

The limitations of the present study should be highlighted in order to provide a more complete understanding of the results and to indicate possible improvements for future investigations. The spatial resolution of Landsat data exceeded that of the climatic variables used in the study, which required a downscaling procedure to reconcile temporal and spatial resolutions. Although downscaling enabled compatibility between variables, this approach may have introduced uncertainties into the results due to data interpolation and potential losses of precision in the climatic variables.

Model accuracy may have been influenced by the predominance of natural vegetation areas, with reduced representation of degraded areas. In the imbalanced dataset, classes with larger sample sizes carried greater weight in the analysis, possibly resulting in lower accuracy for under-represented classes, such as degraded areas, which may have impaired model performance.

Additionally, pixels were treated as independent observations, and spatial autocorrelation was not explicitly modeled in the analysis. This assumption may have led to inflated performance metrics, as neighboring pixels often exhibit high similarity. Future studies could address this limitation by incorporating spatially explicit validation methods to account for spatial dependence and provide more conservative estimates of model performance.

Although the MLR, RF, and KNN models were effective for the analysis, other models might yield improved results. Alternative approaches, such as neural networks, XGBoost, or SVM, could be explored in future research to assess potential gains in predictive accuracy and model capability. The slope variable was not considered among the model predictors but is suggested as a relevant factor to incorporate in future studies. Inclusion of slope could provide a more detailed analysis, particularly in areas with complex relief where slope strongly influences land use dynamics.

This study focused on environmental variables as the principal determinants of land use dynamics. However, socioeconomic factors, such as population density and local infrastructure, also play important roles in LULCC. The absence of these variables in the model limits a comprehensive interpretation of the drivers of LULCC dynamics. Future research should include such socioeconomic variables to enhance understanding of the social and economic processes involved.

## Conclusion

Between 2009 and 2023, analysis of climatic and environmental variables within the Guaraqueçaba EPA revealed distinct patterns of precipitation, temperature, humidity, and land use, with more intense precipitation in the western sector attributable to topography and maritime winds. Maximum temperatures were higher in the southern and southeastern areas, whereas vegetated zones, such as mangroves and the Atlantic Forest, exhibited lower thermal variability and higher evapotranspiration. Solar radiation and relative humidity followed typical tropical patterns, being higher in coastal zones and reduced in anthropized areas. NDVI indicated a relatively stable vegetation cover despite some LULCC, with a general tendency toward conservation within the EPA, albeit under growing pressure from unsustainable practices and shifts in natural resource use.

It is also important to underscore the challenges posed by anthropic occupation and unsustainable practices, such as unregulated extraction of natural resources, introduction of exotic species, and predatory exploitation of fisheries. These factors compromise local biodiversity and ecological stability. Implementation of community-based management practices and adoption of conservation mechanisms are essential to secure environmental protection and the well-being of local communities.

The performance analysis of MLR, KNN, and RF models for land use classification showed significant variations in accuracy, sensitivity, and explanatory capacity depending on the data type (imbalanced versus balanced). The MLR model, although exhibiting an apparent accuracy of 81% in the imbalanced scenario, proved limited in its inability to adequately classify minority classes, with a precision and F1-score of only 72% and a low $R^{2}$ (7.14%), indicating difficulty in capturing data variability. In the balanced scenario, MLR performance declined markedly, accuracy fell to 48%, and prediction error increased substantially, confirming the inadequacy of a linear formulation to represent complex nonlinear relationships in environmental data.

By contrast, KNN demonstrated greater robustness and flexibility, achieving 89% accuracy in the imbalanced scenario and a slight reduction to 86% in the balanced scenario, while maintaining satisfactory precision and F1-score. Variable analysis indicated that KNN is sensitive to climatic drivers such as minimum temperature and is effective at capturing nonlinear interactions.

The RF model distinguished itself as the most efficient: it reached 96% accuracy in the imbalanced scenario, with precision and recall also at 96%, and displayed the lowest MSE (1.96%) and RMSE (14%). In the balanced scenario, RF maintained 95% accuracy and an $R^{2}$ of 88.6%, demonstrating superior capacity to adapt to balanced data distributions.

Identification of the most relevant predictors, notably precipitation and NDVI, reflects the pivotal role of these factors in local ecological dynamics. Analysis of their influence on land use can inform conservation strategies focused on preserving high vegetation cover areas and zones most sensitive to climatic variability, such as regions with high precipitation and dense vegetation. This is particularly relevant given the crucial role of water and vegetation in maintaining the resilience of ecosystems within the Guaraqueçaba EPA, especially in the face of environmental pressures and resource exploitation.

The results further suggest that ML models, such as RF, constitute valuable tools for evaluating the effectiveness of conservation strategies over time and for identifying priority areas for restoration and protection. Continuous application of these models would enable monitoring of land use evolution and rapid adjustment of management policies to respond to environmental changes, including vegetation loss or increased anthropic pressure.

From an environmental management perspective, these findings highlight the need to integrate predictive models and environmental data into strategic planning so that decision-making is grounded in detailed, up-to-date information. Deployment of such technological tools can also support the development of more effective policies for the recovery of degraded areas and the mitigation of anthropogenic impacts, thereby ensuring ecosystem protection and the long-term sustainability of the Guaraqueçaba EPA.

## Supplementary Information

Below is the link to the electronic supplementary material.

Supplementary file 1 (pdf 68 KB)

## Data Availability

The data used to support the findings of this study are available from the corresponding author upon request.
