# Supplementary material for: Machine learning for land use change analysis in environmental protection areas
Source: Environ Monit Assess. 2026 Apr 20;198(5):481. doi: 10.1007/s10661-026-15280-7 (PMC13095965; doi:10.1007/s10661-026-15280-7)
Supplement: Supplementary file 1 — (pdf 68 KB) [file 10661_2026_15280_MOESM1_ESM.pdf]

Supplementary material

Year	Date
2009	24/05
	05/03
2010	04/02
	19/11
2011	12/04
	02/08
2012	06/04
	21/03
2013	30/07
	11/11
2014	30/01
	01/07
2015	29/08
	09/01
2016	12/06
	11/11
2017	14/05
	14/01
2018	12/07
	11/12
2019	23/07
	28/01
2020	04/04
	13/10
2021	25/05
	14/03
2022	13/06
	27/10
2023	15/05
	03/08
